# Supplementary material for: Gαi2-induced conductin/axin2 condensates inhibit Wnt/β-catenin signaling and suppress cancer growth
Source: Nat Commun. 2022 Feb 3;13:674. doi: 10.1038/s41467-022-28286-9 (PMC8814139; doi:10.1038/s41467-022-28286-9)
Supplement: Supplementary file 1 — Supplementary Information [file 41467_2022_28286_MOESM1_ESM.pdf]

Supplementary Information

Supplementary Fig. 1

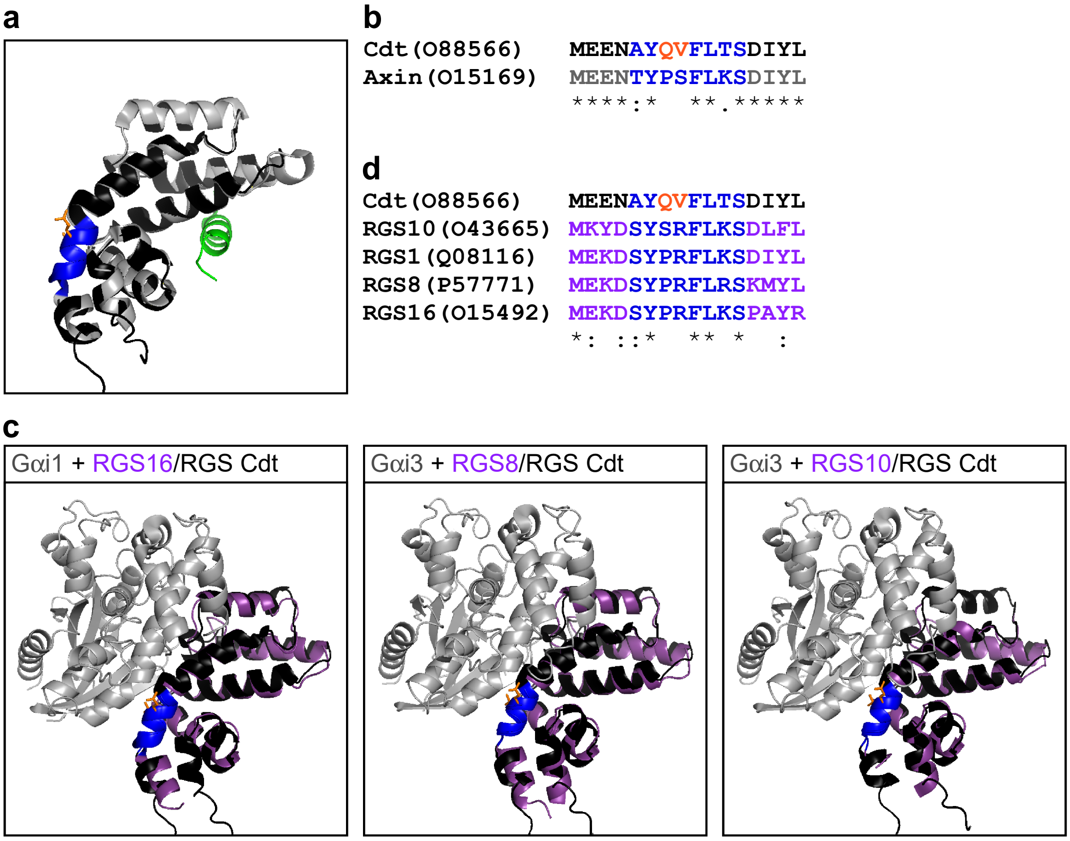

Supplementary Fig. 1 Structural models for binding of RGS interactors to the conductin RGS domain. **a**, **c** Structural alignment of the modelled conductin RGS domain (black) to the axin RGS domain (grey) co-crystalized with an APC SAMP repeat (green; 1EMU; **a**), to RGS16 (purple) co-crystalized with Gαi1 (grey; 2IK8), to RGS8 (purple) co-crystalized with Gαi3 (grey; 2ODE) and to RGS10 (purple) co-crystalized with Gαi3 (grey; 2IHB; **c**)[1]. **b**, **d** Clustal Omega alignment of conductin (Cdt), axin, RGS1, RGS8, RGS10 and RGS16 protein sequences around the aggregon that had been identified in the Cdt RGS domain[2]. UniProt entry identifiers are shown in brackets. Identity (*) and conservation between amino acid groups of strongly (:) and weakly (.) similar properties are indicated[3]. **a**-**d** The position of the conductin aggregon and its two key residues glutamine and valine[2] are highlighted in blue and orange, respectively.

Supplementary Fig. 2

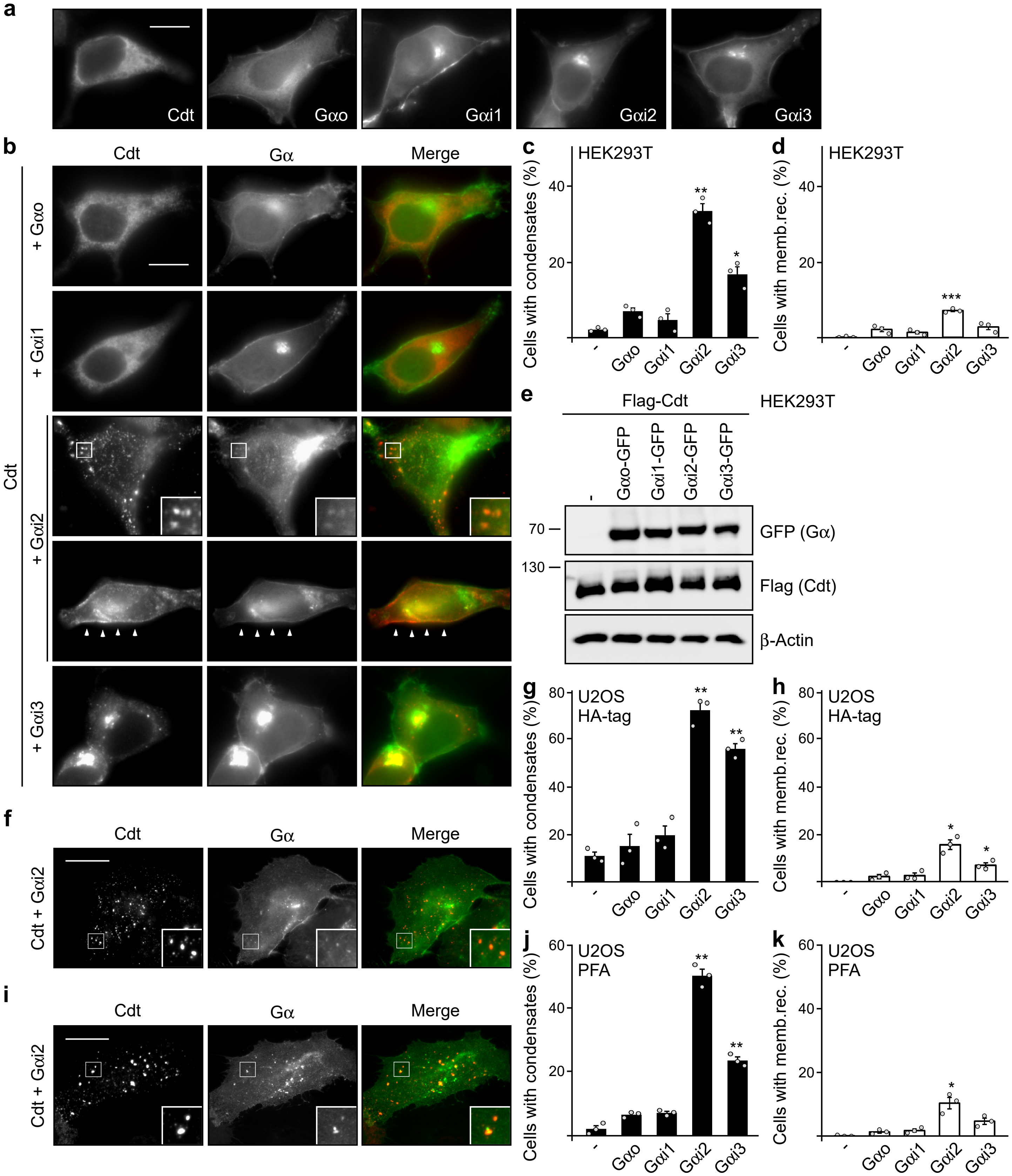

Supplementary Fig. 2 Gαi2 induces polymerization of conductin. **a**, **b**, **f**, **i** Immunofluorescence staining (red) of Flag (**a**, **b**, **i**) or HA (**f**) in HEK293T cells transfected with Flag-Conductin (Cdt) and different GFP-tagged Gα proteins (green) either alone (**a**) or in indicated combinations (**b**), and in U2OS cells transfected with HA-Conductin (**f**) or Flag-Conductin (**i**) together with Gαi2-GFP. Cells in **i** were fixed with PFA in contrast to methanol fixation of the other experiments. Scale bars: 10 µm (**a**, **b**) and 20 µm (**f**, **i**). Insets are magnified in the lower right corner. Arrowheads point to membrane recruitment. **c**, **d**, **g**, **h**, **j**, **k** Percentage of 900 transfected cells out of three independent experiments as in **b**, **f** or **i** showing condensation (**c**, **g**, **j**) or membrane recruitment (**d**, **h**, **k**) of conductin. Results are mean +/- SEM (n=3). *p<0.05, **p<0.01, ***p<0.001 (Two-sided Student's *t*-test). **e** Western blotting for GFP, Flag and β-actin (loading control) in lysates of HEK293T cells transfected with indicated constructs. Molecular weight is indicated in kDa. Source data and exact p-values are provided as source data file.

Supplementary Fig. 3

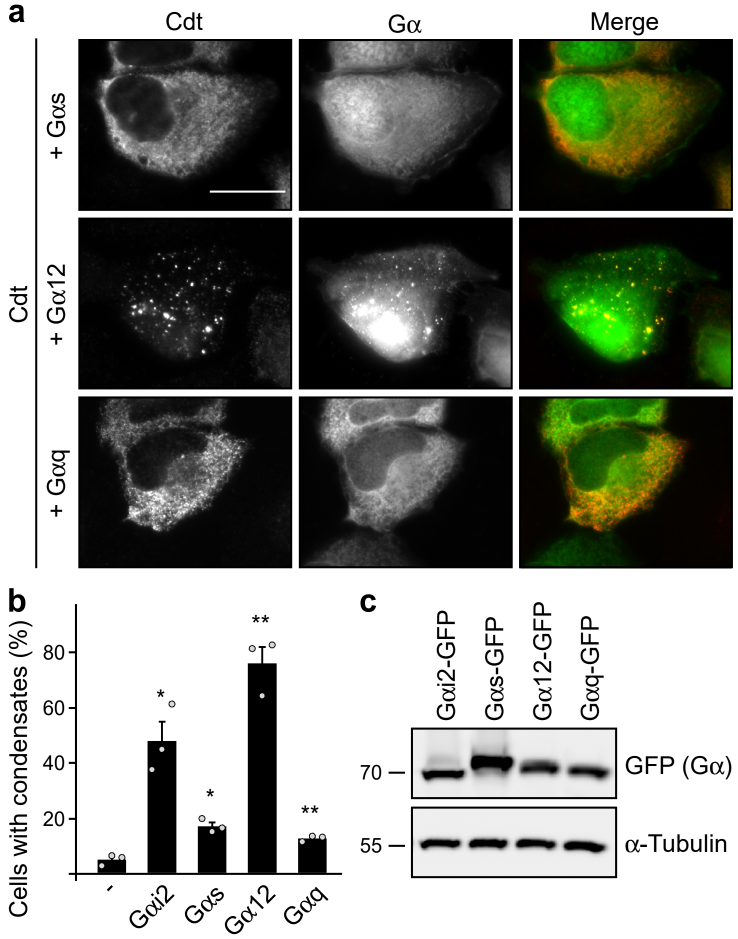

Supplementary Fig. 3 Conductin polymerization by Gα proteins from other families.

a Immunofluorescence staining of Flag (red) in U2OS cells transfected with Flag-Conductin (Cdt) together with indicated GFP-tagged Gα proteins (green). Scale bar: 20 µm.

b Percentage of 900 transfected cells out of three independent experiments as in **a** showing condensation of conductin. Results are mean +/- SEM (n=3). *p<0.05, **p<0.01 (Two-sided Student's *t*-test). **c** Western blotting for GFP and α-tubulin (loading control) in lysates of U2OS cells transfected as indicated, showing similar expression of the Gα proteins used in **a**. Molecular weight is indicated in kDa. Source data and exact p-values are provided as source data file.

Supplementary Fig. 4

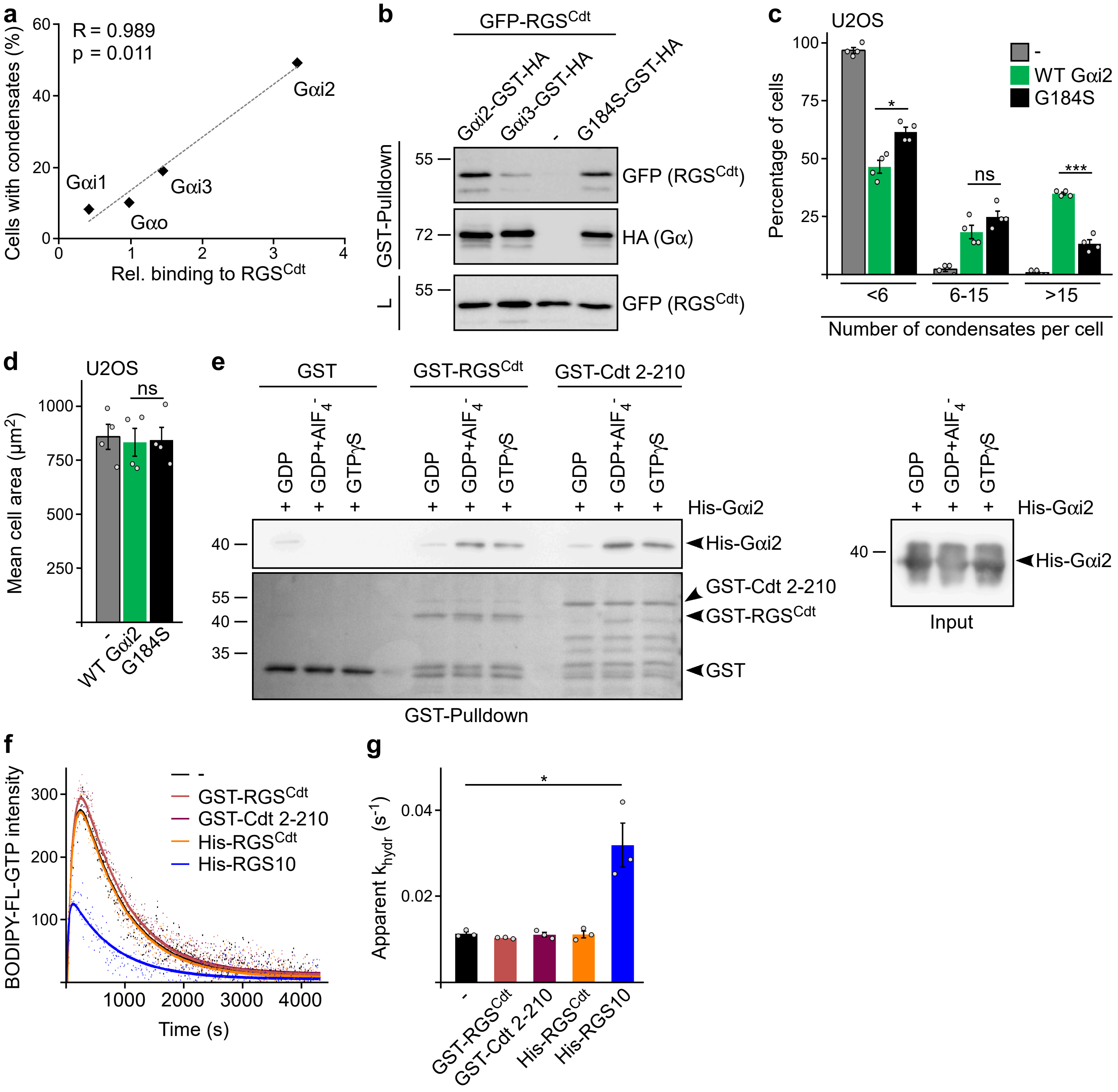

Supplementary Fig. 4 Induction of conductin polymerization positively correlates with Gαi2-RGS binding. **a** Plotting of conductin condensates induction (Fig. 1d) against RGS domain binding (Fig. 2b) for indicated Gα proteins. R: Pearson‘s correlation coefficient, p: p-value.
b Western blotting for GFP and HA in lysates (L) of HEK293T cells transfected with indicated constructs, and after GST-pulldown from these lysates. G184S is a Gαi2 point mutant.
c Percentage of cells with less than 6, between 6 and 15, or more than 15 conductin condensates per cell out of 200 transfected U2OS cells from four independent experiments similarly performed as in Fig. 1c. Number of condensates per cell was determined via image-based analysis using the Icy Spot Detector tool (see Methods). **d** Mean area of the cells analyzed in **c**, showing similar sizes of the investigated cells. **e** Left: Pulldown of purified recombinant GST, GST-RGS^{Cdt} and a N-terminal fragment of conductin containing the RGS domain (GST-Cdt 2-210) after incubation with purified His-Gαi2, which was preloaded with GDP, GDP+AlF_4^- or GTPγS. Western blot and the Ponceau stained gel before blotting show co-precipitated His-Gαi2 and precipitated GST proteins, respectively. Right: Western blotting showing equal input of GDP-, GDP+AlF_4^-- and GTPγS-loaded His-Gαi2 for the pulldown experiment. **f** Time course analysis of fluorescence intensity (arbitrary units) of the GTP derivative BODIPY-FL-GTP, which was incubated with purified Gαi2 alone (-) or together with indicated RGS domain-containing proteins. BODIPY-FL-GTP fluorescence increases due to Gαi2 binding, and decreases due to hydrolysis by the GTPase activity of Gαi2. **g** Rate constant of BODIPY-FL-GTP hydrolysis (apparent k_{hydr}) by Gαi2 in the presence of indicated RGS proteins calculated from three independent experiments as in **f**. Note that the conductin RGS domain did not promote the GTPase activity of Gαi2. The Gαi GAP RGS10 functions as positive control[4]. Results are mean +/- SEM (n=4 [**c**, **d**], n=3 [**g**]). *p<0.05, ***p<0.001 (Two-sided Student’s *t*-test). Molecular weight is indicated in kDa (**b**, **e**). Source data and exact p-values are provided as source data file.

Supplementary Fig. 5

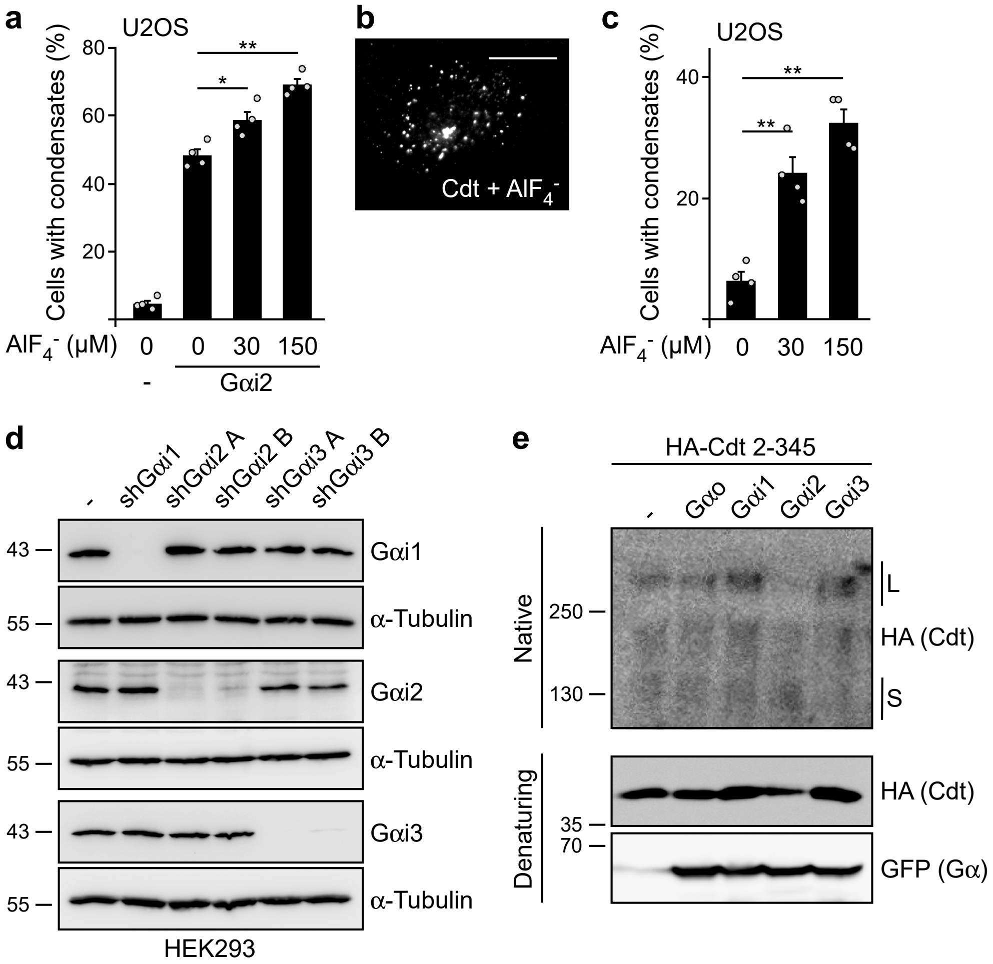

Supplementary Fig. 5 AlF_4^- treatment promotes conductin condensation. **a**, **c** Percentage of cells showing conductin condensation out of 1200 transfected U2OS cells from four independent experiments, which were transfected with Flag-Conductin either together with Gαi2-GFP (**a**) or alone (**c**), and treated with indicated AlF_4^- concentrations for 1 h. Results are mean +/- SEM (n=4). *p<0.05, **p<0.01 (Two-sided Student's *t*-test). **b** Immunofluorescence staining of Flag-Conductin (Cdt) in AlF_4^- treated U2OS cells. Scale bar: 20 µm. **d** Western blotting for Gαi1, Gαi2, Gαi3 and α-tubulin (loading control) in lysates of HEK293 cells stably transfected with indicated shRNAs. One out of three representative experiments is shown.
e Alternative Western blot to Fig. 2i of a similarly performed experiment. Note the decrease and increase of large (L) and small (S) complexes by Gαi2, respectively. One out of three representative experiments is shown. Molecular weight is indicated in kDa (**d**, **e**). Source data and exact p-values are provided as source data file.

Supplementary Fig. 6

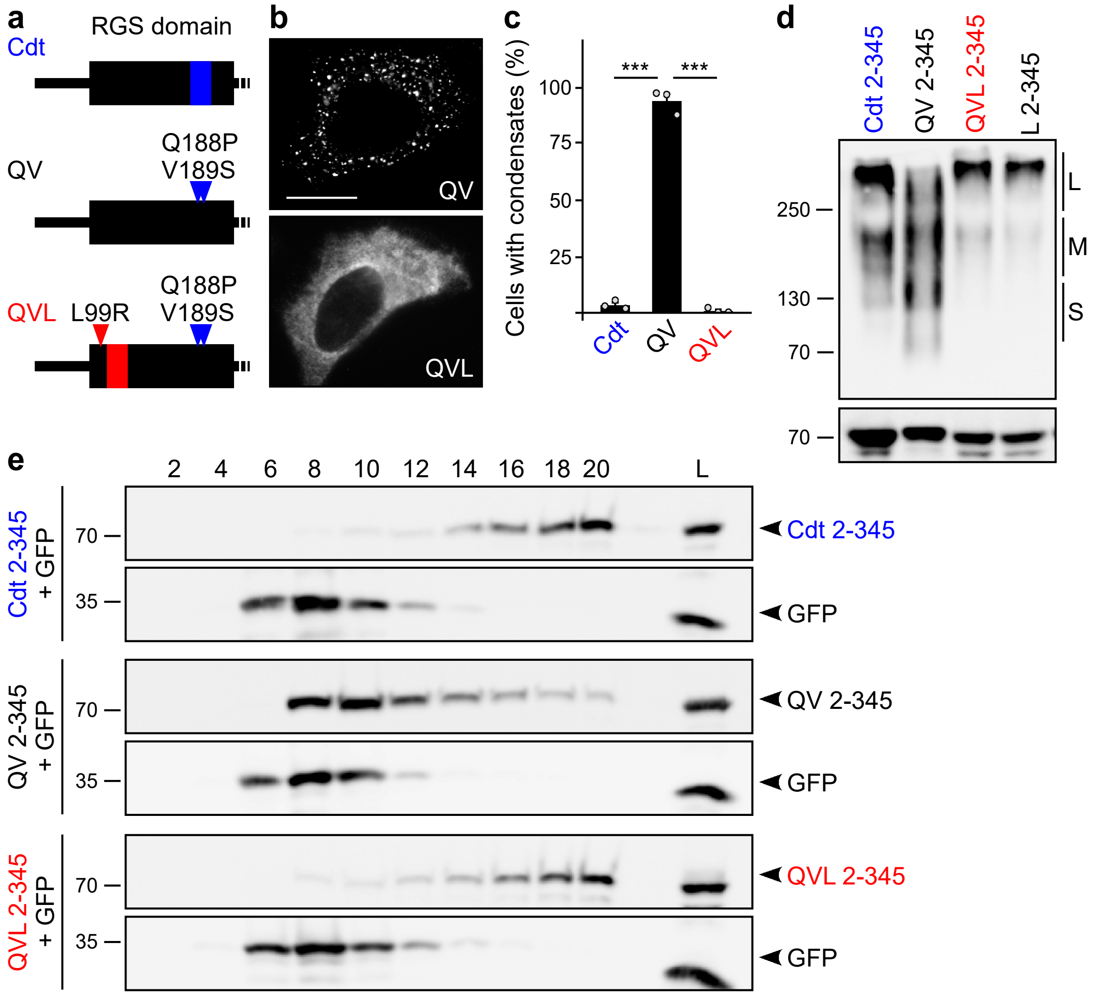

Supplementary Fig. 6 Activation of a silent aggregation site can compensate for inactivation of the aggregon. **a** Schematic representation of the RGS domain of WT conductin (Cdt), the QV and the QVL mutant. Active aggregation sites are indicated by blue and red boxes. Blue and red arrowheads indicate point mutations inactivating the blue aggregon (Q188P; V189S) or activating the red aggregation site (L99R), respectively. The L99R mutation corresponds to the axin L106R mutation, a mutation detected in liver cancer that activates an otherwise silent aggregation site[5]. **b** GFP fluorescence in U2OS cells expressing indicated GFP-tagged conductin mutants. Scale bar: 20 µm. **c** Percentage of 900 transfected cells out of three independent experiments as in **b** showing condensation of the indicated constructs. Results are mean +/- SEM (n=3). ***p<0.001 (Two-sided Student‘s *t*-test). **d** Western blotting under native (upper panel) and denaturing (lower panel) conditions for GFP in lysates of U2OS cells transfected with GFP-tagged indicated constructs. Large (L), medium (M) and small (S) aggregation complexes are indicated at the right. **e** Western blotting for GFP-tagged WT conductin 2-345, its QV and QVL mutants, and GFP in lysates (L) of HEK293T cells transfected as indicated on the left, and after fractionation of these lysates via ultracentrifugation through a sucrose density gradient (sucrose density increases from fraction 2 to 20). Molecular weight is indicated in kDa (**d**, **e**). **a**-**e** Color indicates aggregation via the blue aggregon or the red aggregation site highlighted in Fig. 3b. Source data and exact p-values are provided as source data file.

Supplementary Fig. 7

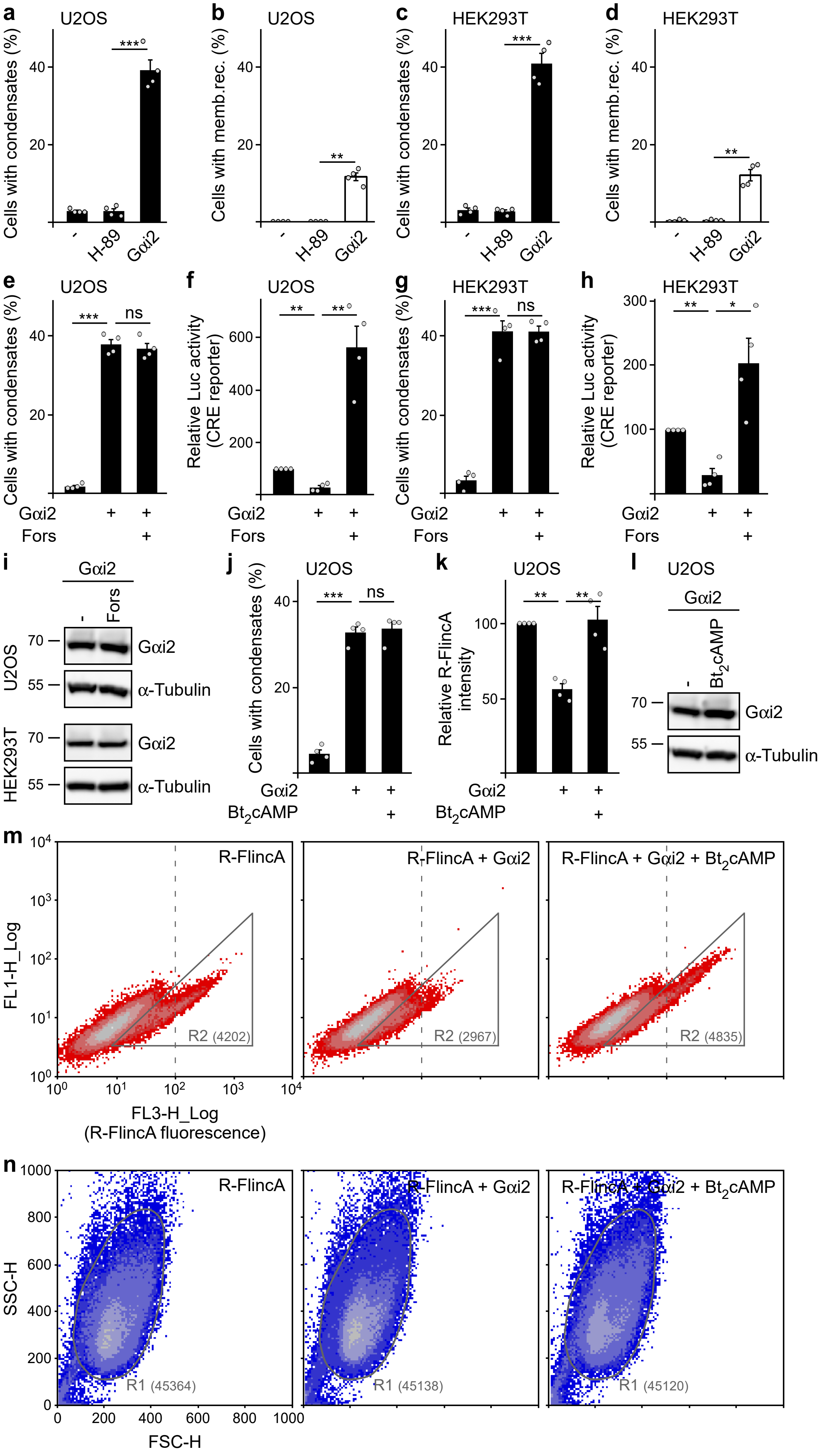

Supplementary Fig. 7 Conductin condensates form independent of Gαi2-cAMP-PKA signaling. **a**-**e**, **g**, **j** Percentage of transfected cells showing Flag-Conductin condensates (**a**, **c**, **e**, **g**, **j**) or membrane recruitment (**b**, **d**) of U2OS (**a**, **b**) or HEK293T cells (**c**, **d**) that were untreated, treated with 30 µM H-89 or transfected with 300 ng Gαi2, and of U2OS (**e**, **j**) or HEK293T cells (**g**) that were transfected with Gαi2 without and with 20 µM forskolin (Fors) or 1 mM Bt_2cAMP treatment overnight, as indicated. Per bar 1200 transfected cells from four independent experiments were analyzed. **f**, **h** Luciferase activity (CRE reporter) in U2OS (**f**) and HEK293T cells (**h**) that were transfected and treated similarly and in parallel to the immunofluorescence experiments in **e** and **g**, respectively. **i**, **l** Western blotting for Gαi2-GFP in Gαi2 transfected U2OS or HEK293T cells without and with 20 µM forskolin or 1 mM Bt_2cAMP treatment overnight, as indicated, showing that Gαi2 expression levels are treatment independent. α-Tubulin: loading control. Molecular weight is indicated in kDa. **k** FACS-based quantification of R-FlincA fluorescence intensity in U2OS cells that were transfected and treated similarly and in parallel to the immunofluorescence experiments in **j**, from four independent experiments. Results are mean +/- SEM (n=4). *$p<0.05$, **$p<0.01$, ***$p<0.001$ (Two-sided Student's *t*-test). **m** FACS analysis of R-FlincA fluorescence intensity of one representative experiment quantified in **k**. R2 labels the region of the red fluorescent R-FlincA positive cells (FL3 intensity [red] distinctly higher than FL1 intensity [green = auto fluorescence of the cells]), which were used for quantification. Number of cells within R2 is provided in brackets. **n** Gating based on forward scatter (FSC) and side scatter (SSC) to exclude cell debris. R1 labels the region of the cells that were included in the analysis shown in **m**, which were about 90% of the 50,000 sorted cells; the exact cell number is provided in brackets. Source data and exact p-values are provided as source data file.

Supplementary Fig. 8

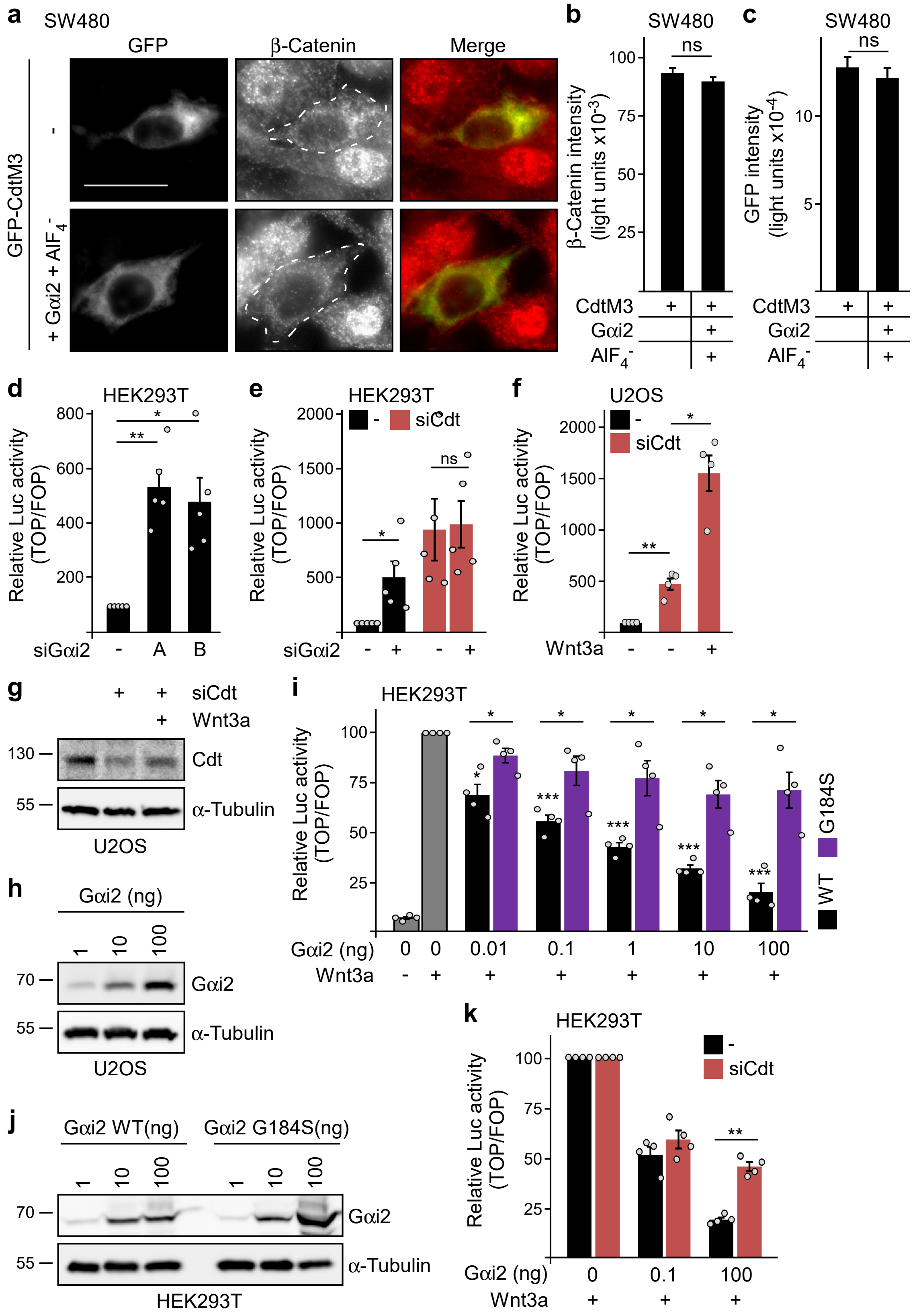

Supplementary Fig. 8 Gαi2 inhibits Wnt signaling via conductin. **a** Immunofluorescence staining of endogenous β-catenin (red) in SW480 cells, which were transfected and treated as indicated on the left. Scale bar: 20 µm. **b**, **c** Quantification of β-catenin (**b**) and GFP fluorescence intensities (**c**) in four independent experiments as in **a**. **d**, **e** Luciferase activity (TOP/FOP) in HEK293T cells which were transfected with different siRNAs (A, B) against Gαi2 (**d**) or transfected with indicated combinations of a control siRNA (-), siGαi2 and siCdt (**e**).
f Luciferase activity (TOP/FOP) in U2OS cells which were transfected with a control siRNA or siCdt and treated with Wnt3a, as indicated. **g** Western blotting showing conductin knock-down (and Wnt3a-induced partial re-expression) in the U2OS cells used in **f**. α-Tubulin: loading control. **h**, **j** Western blotting showing dosage dependent expression of WT Gαi2 in U2OS cells (**h**) and of WT Gαi2 and the G184S mutant in HEK293T cells (**j**). α-Tubulin: loading control. Molecular weight is indicated in kDa. **i**, **k** Luciferase activity (TOP/FOP) in HEK293T cells which were transfected with rising amounts of WT Gαi2 or the G184S mutant without and with Wnt3a treatment (**i**) or transfected with indicated Gαi2 amounts together with a control siRNA or siCdt and treated with Wnt3a (**k**). To facilitate comparisons of the Gαi2 effects in cells with (black bars) and without conductin (siCdt, red bars) in **k**, the initial luciferase activities without Gαi2 were set to 100% for both conditions, and the luciferase activities with Gαi2 are presented relative to the respective initial activity. Results are mean +/- SEM (n=80 [**b**, **c**], n=5 [**d**, **e**], n=4 [**f**, **i**, **k**]). *p<0.05, **p<0.01, ***p<0.001 (Two-sided Student's *t*-test). Source data and exact p-values are provided as source data file.

Supplementary Fig. 9

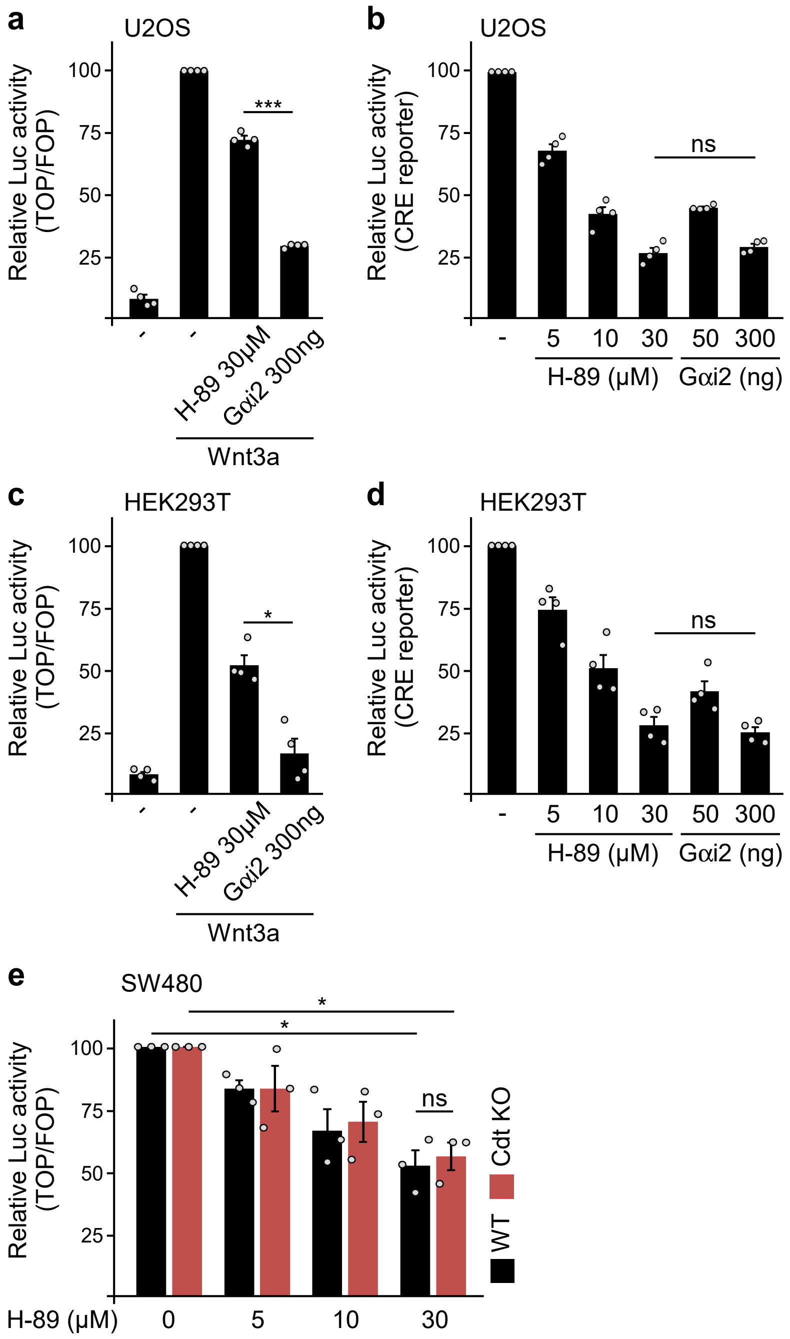

Supplementary Fig. 9 Gαi2 inhibits Wnt signaling more potently than direct PKA inhibition. **a**-**d** Luciferase activity reporting β-catenin signaling (TOP/FOP, **a**, **c**) or PKA activity (CRE reporter, **b**, **d**) in U2OS (**a**, **b**) and HEK293T cells (**c**, **d**), which were treated with H-89 and/or Wnt3a, or transfected with Gαi2, as indicated. TOP/FOP and CRE reporter experiments were always performed in parallel. **e** Luciferase activity (TOP/FOP) in SW480 WT and *AXIN2/Conductin* knockout (Cdt KO) cells which were treated with indicated H-89 concentrations. To facilitate comparisons of the H-89 effects in cells with (black bars) and without conductin (red bars), the initial luciferase activities without H-89 were set to 100% for both conditions, and the luciferase activities after H-89 treatment are presented relative to the respective initial activity. Results are mean +/- SEM (n=4 [**a**-**d**], n=3 [**e**]). *p<0.05, ***p<0.001 (Two-sided Student's *t*-test). Source data and exact p-values are provided as source data file.

Supplementary Fig. 10

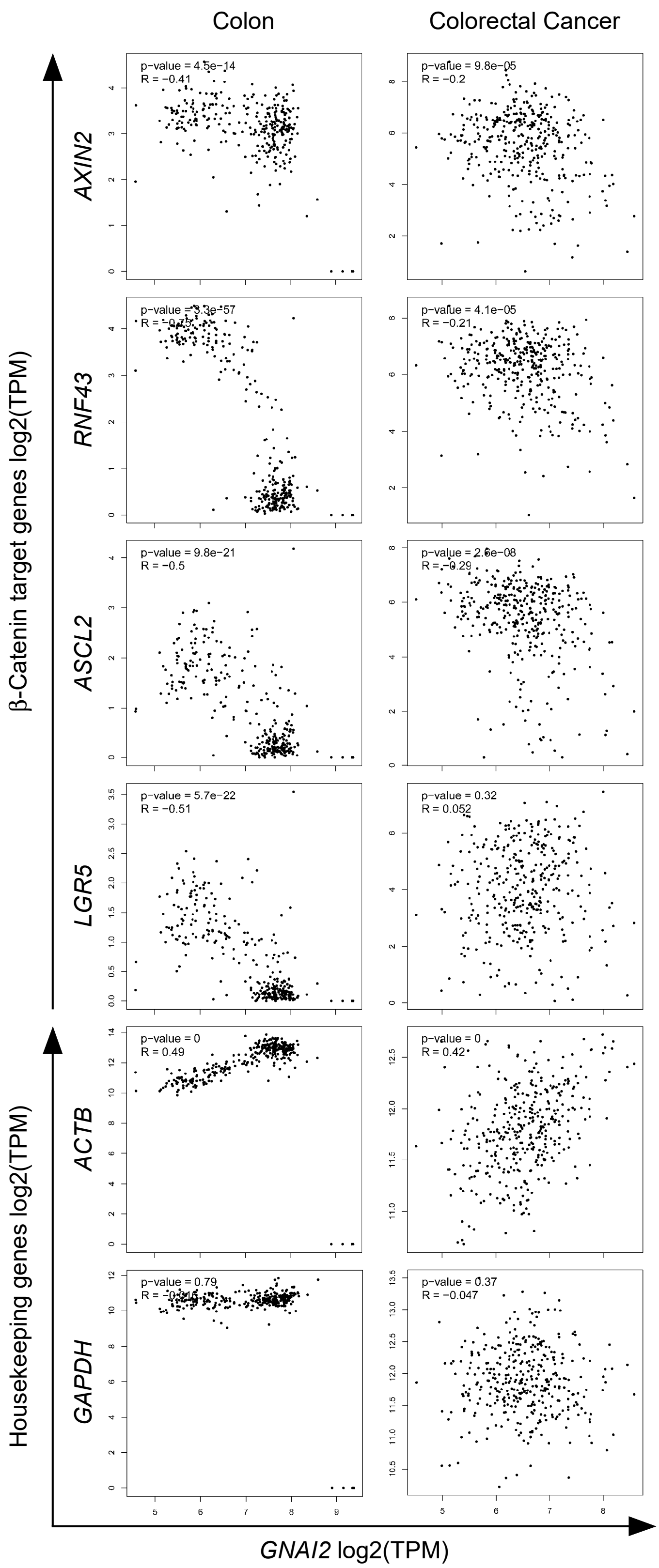

Supplementary Fig. 10 Expression of Gαi2 and β-catenin target genes negatively correlates. Plotting of *GNAI2* (Gαi2) mRNA expression against expression of indicated β-catenin target genes and housekeeping genes in colon tissue samples (left column) and colorectal cancer samples (right column). R: Pearson's correlation coefficient. TPM: transcripts per million. The analysis was performed by GEPIA using expression data from The Genotype-Tissue Expression (GTEx) and the TCGA colorectal cancer datasets COAD and READ[6, 7, 8].

Supplementary Fig. 11

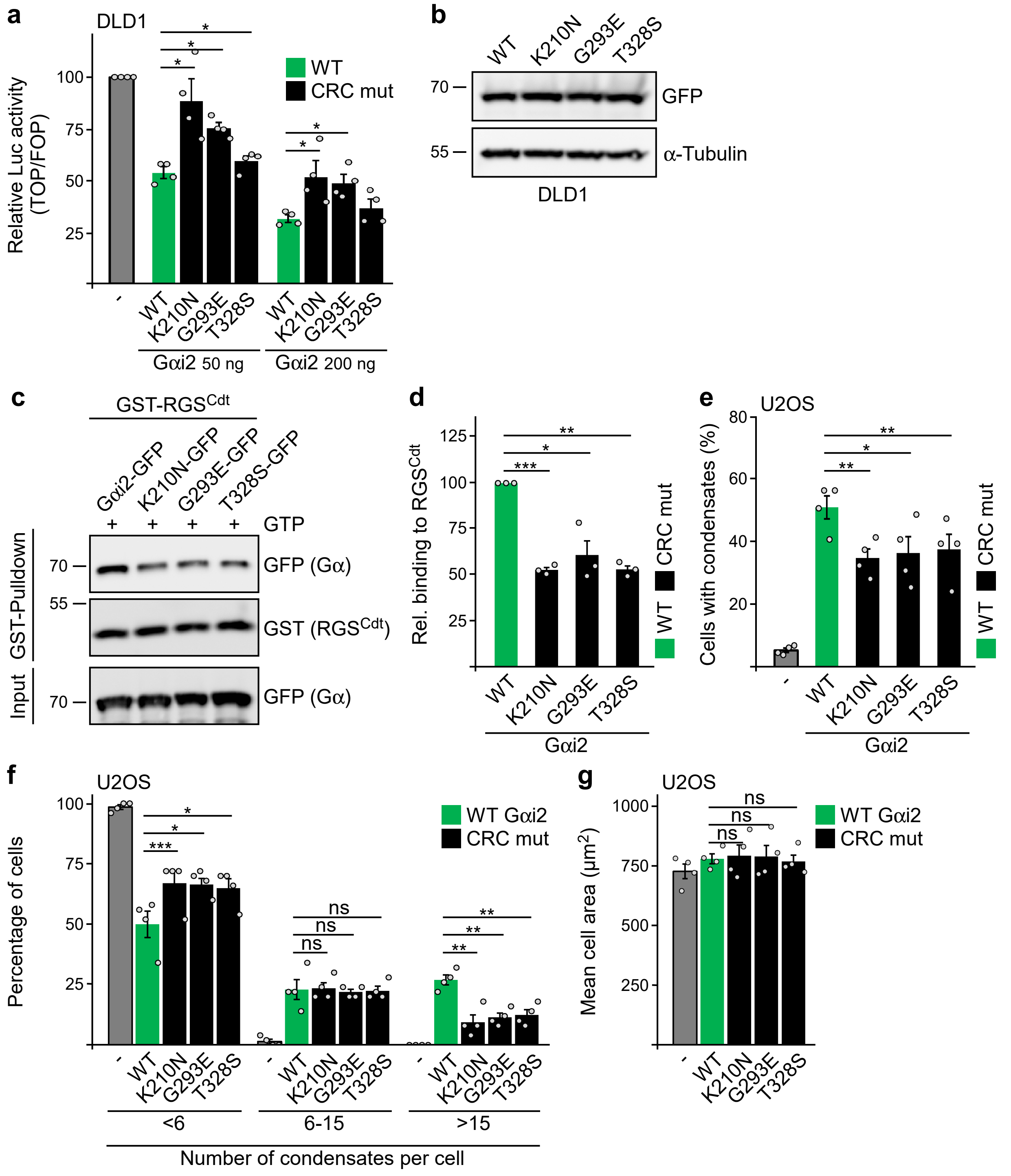

Supplementary Fig. 11 Gαi2 cancer mutations impair Wnt signaling inhibition and conductin regulation.
a Luciferase activity (TOP/FOP) in DLD1 colorectal cancer cells transfected with rising amounts of WT Gαi2-GFP or indicated colorectal cancer mutants (CRC mut). **b** Western blotting showing equal expression of Gαi2-GFP WT and colorectal cancer mutants in DLD1 cells. α-Tubulin: loading control. **c** Western blotting for GFP and GST after GST-pulldown of purified recombinant GST-RGS^{Cdt} that was incubated with indicated in vitro synthesized Gαi2-GFP proteins, and Western blotting for GFP showing equal synthesis of the Gαi2-GFP proteins used for the pulldown experiment (Input). Molecular weight is indicated in kDa. **d** Quantification of the amounts of indicated Gαi2-GFP proteins that were co-precipitated with GST-RGS^{Cdt} in three independent experiments as in **c**, presented relative to WT.
e Percentage of cells with conductin condensates out of 1200 U2OS cells transfected with Flag-tagged conductin either alone, with WT Gαi2-GFP or with indicated mutants, from four independent experiments similarly performed as in Fig. 1c. **f** Percentage of cells with less than 6, between 6 and 15, or more than 15 conductin condensates per cell out of 200 transfected U2OS cells from four independent experiments performed as in **e**. Number of condensates per cell was determined via image-based analysis using the Icy Spot Detector tool (see Methods). **g** Mean area of the cells analyzed in **f**, showing similar sizes of the investigated cells. Results are mean +/- SEM (n=4 [**a**, **e**, **f**, **g**], n=3 [**d**]). *p<0.05, **p<0.01, ***p<0.001 (Two-sided Student's *t*-test). Source data and exact p-values are provided as source data file.

Supplementary Fig. 12

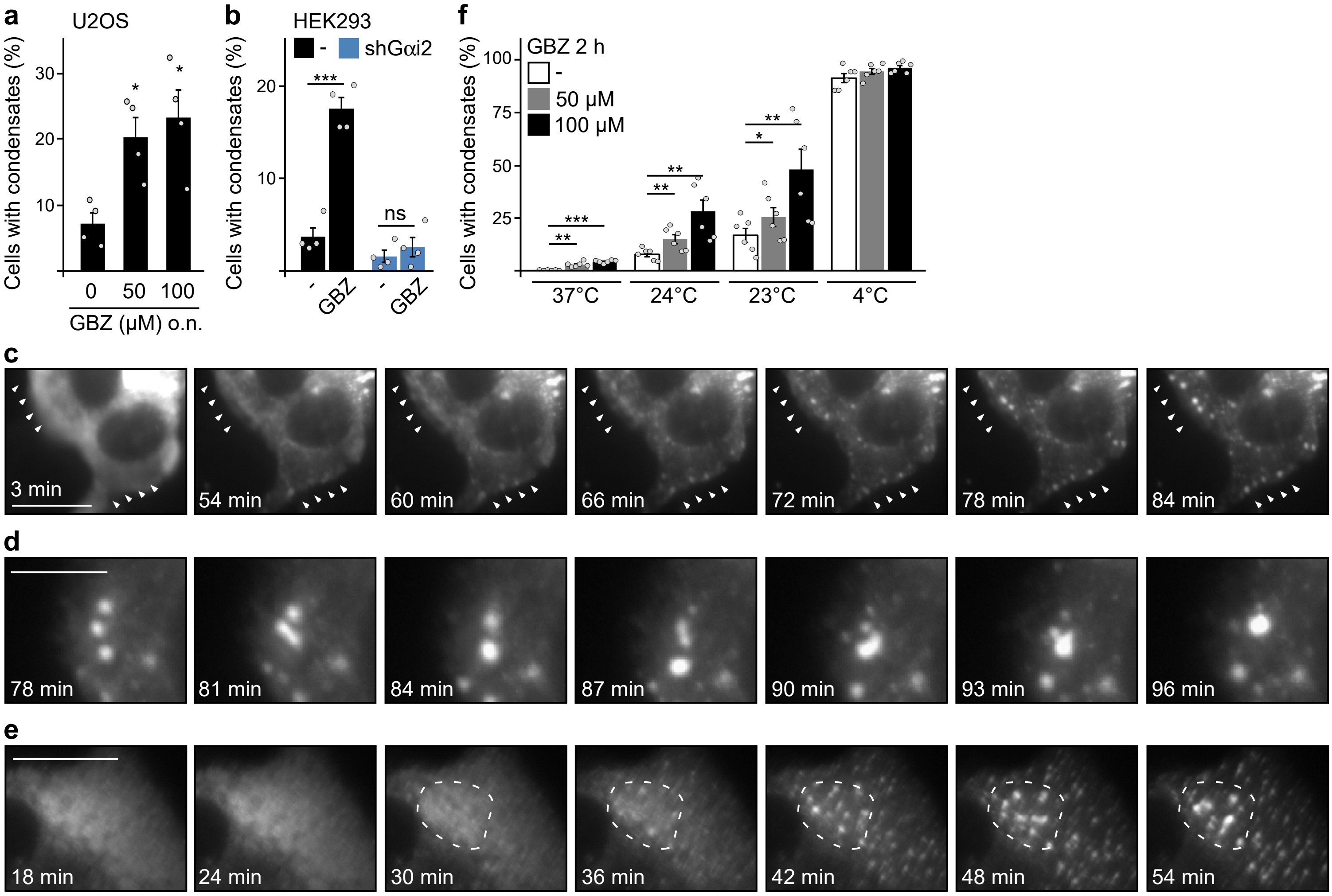

Supplementary Fig. 12 GBZ treatment triggers conductin condensation. **a** Percentage of cells with conductin condensates out of 1200 U2OS cells transfected with HA-tagged conductin without and with GBZ treatment from four independent experiments similarly performed as in Fig. 6a. **b** HEK293 cells stably expressing either a control shRNA (-) or a Gαi2-targeting shRNA were transfected with HA-Conductin, and left untreated or treated o.n. with 100 µM GBZ. Percentage of cells with conductin condensates out of 800 transfected cells from four independent experiments is shown. **c**-**e** GFP fluorescence in U2OS cells stably expressing GFP-Conductin after treatment with 100 µM GBZ for the indicated times. Arrowheads point to condensates forming beneath plasma membranes (**c**), and an area with high local conductin concentration is encircled in **e**. Scale bars: 20 µm (**c**, **e**) and 10 µm (**d**).
f Percentage of cells with conductin condensates out of 1800 U2OS cells stably expressing GFP-Conductin, which were treated with 0, 50 and 100 µM GBZ for 2 h at the indicated temperatures. Results are mean +/- SEM (n=4 [**a**, **b**], n=6 [**e**]). *p<0.05, **p<0.01, ***p<0.001 (Two-sided Student's *t*-test). Source data and exact p-values are provided as source data file.

Supplementary Fig. 13

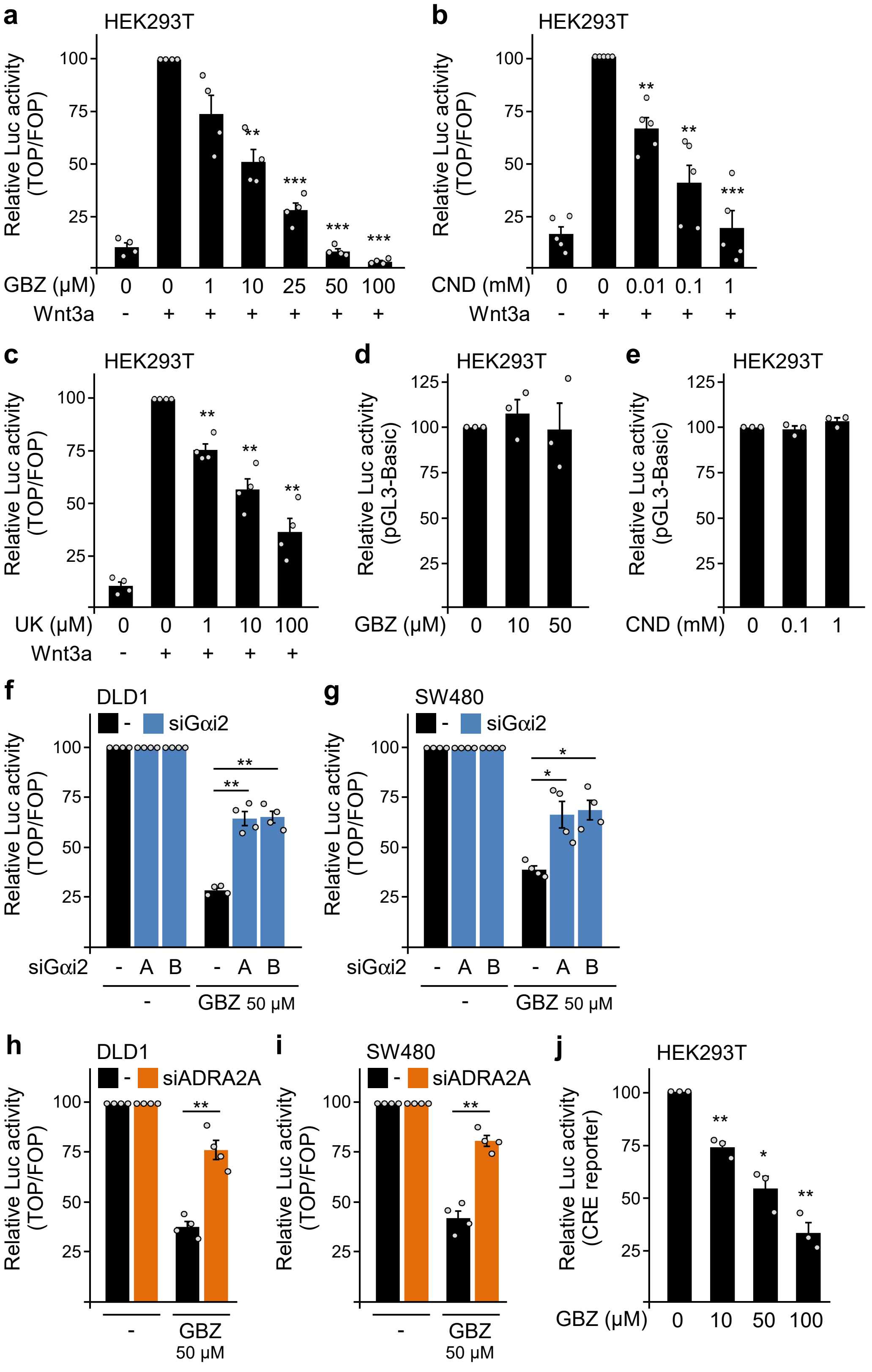

Supplementary Fig. 13 α2-Adrenoceptor agonists inhibit Wnt signaling via Gαi2.
a-**i** Luciferase activity reporting β-catenin signaling (TOP/FOP, **a**-**c** and **f**-**i**) or of a β-catenin-independently transcribed luciferase (pGL3-Basic, **d**, **e**) in HEK293T cells, which were treated with Wnt3a and the α2-adrenoceptor agonists guanabenz (GBZ), clonidine (CND) and UK 14,304 (UK), as indicated (**a**-**e**), and in untreated or GBZ treated DLD1 (**f**, **h**) and SW480 cells (**g**, **i**) transfected with a control siRNA, one of two different siRNAs targeting Gαi2 or a siRNA pool targeting the α2a-adrenoceptor (ADRA2A). **j** Luciferase activity reporting PKA activity (CRE reporter) in HEK293T cells, which were treated with indicated GBZ concentrations. Results are mean +/- SEM (n=4 [**a**, **c**, **f**-**i**], n=5 [**b**], n=3 [**d**, **e**, **j**]). *p<0.05, **p<0.01, ***p<0.001 (Two-sided Student's *t*-test). Source data and exact p-values are provided as source data file.

Supplementary Fig. 14

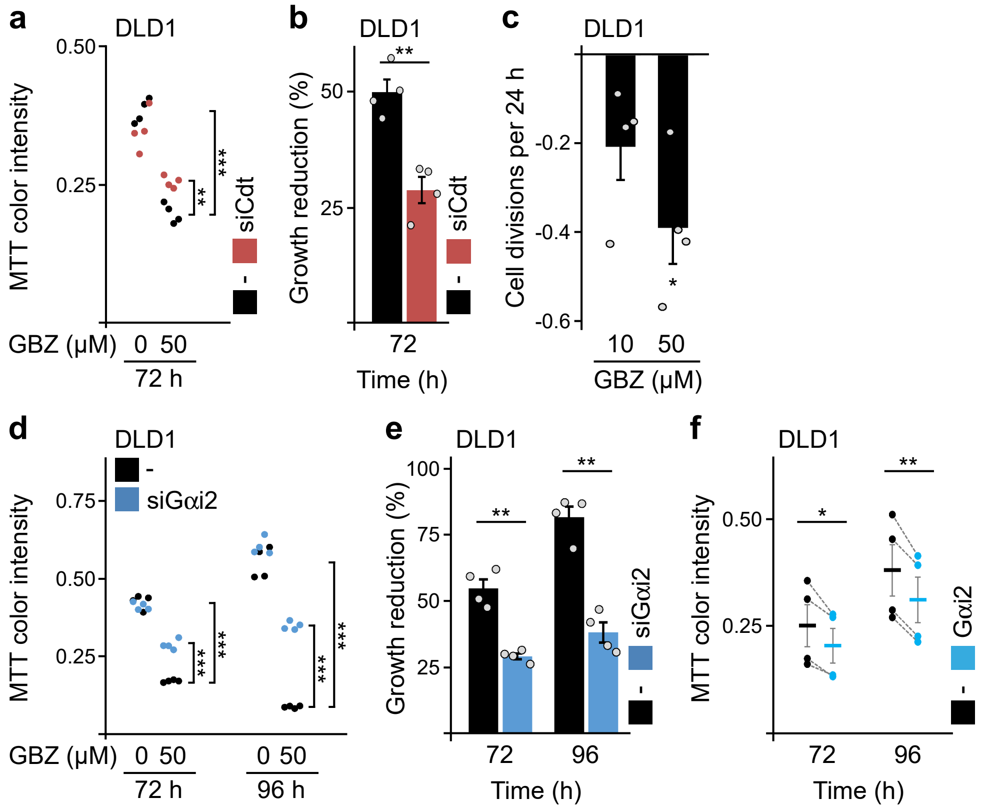

Supplementary Fig. 14 GBZ treatment inhibits growth of DLD1 colorectal cancer cells. **a**, **d** MTT color intensity reflecting the number of viable DLD1 cells growing for 72 or 96 h in the presence of 0 and 50 µM GBZ. Control siRNA transfected cells (-) were compared to cells with conductin knockdown (siCdt, **a**) or to cells with Gαi2 knockdown (siGαi2, **d**). Each dot represents a technical replicate (n=4). **b**, **e** Growth reduction by GBZ of four independent experiments as in **a** (**b**) and as in **d** (**e**) (growth reduction is calculated as the difference between the MTT color intensities without and with GBZ treatment, and presented as percentage relative to the MTT intensities without treatment). **c** Reduction of cell divisions of DLD1 cells treated with indicated GBZ concentrations compared to untreated cells, calculated based on four independent CFSE dilution experiments as shown in Supplementary Fig. 15. **f** MTT color intensity reflecting the number of viable DLD1 cells expressing similar levels of GFP (-, control) or Gαi2-GFP after cell sorting and growth for 72 and 96 h (see Methods for details). The dots represent independent experiments (n=4), and the lines connect values of GFP and Gαi2-GFP expressing cells of the same experiment. Results are mean +/- SEM (n=4). *p<0.05, **p<0.01, ***p<0.001 (Two-sided Student's *t*-test). Source data and exact p-values are provided as source data file.

Supplementary Fig. 15

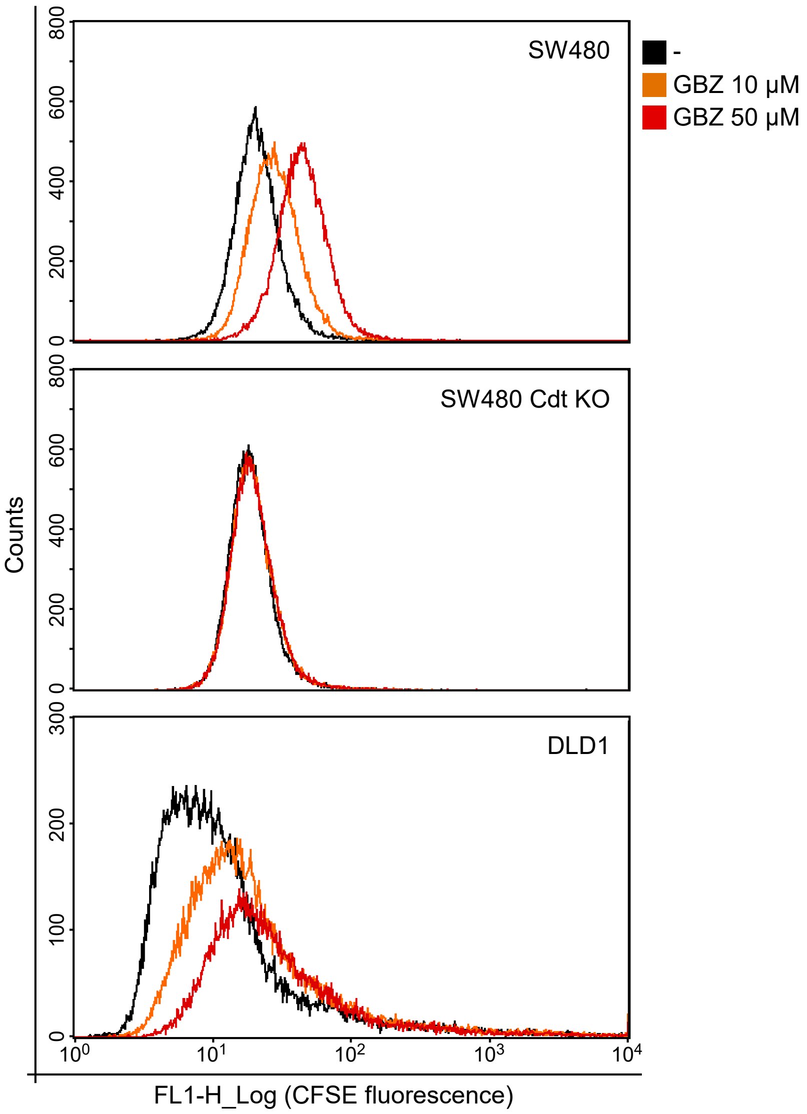

Supplementary Fig. 15 GBZ treatment reduces proliferation of colorectal cancer cells via conductin. FACS-measured remaining carboxyfluorescein succinimidyl ester (CFSE) fluorescence intensity in SW480 WT and *AXIN2/Conductin* knockout cells (Cdt KO), and DLD1 cells that were pulse labelled with CFSE and cultured for 72 h in the presence of indicated GBZ concentrations. Every cell division reduces the CFSE staining intensity by 50%, since it is equally distributed between the two daughter cells.

Supplementary Fig. 16

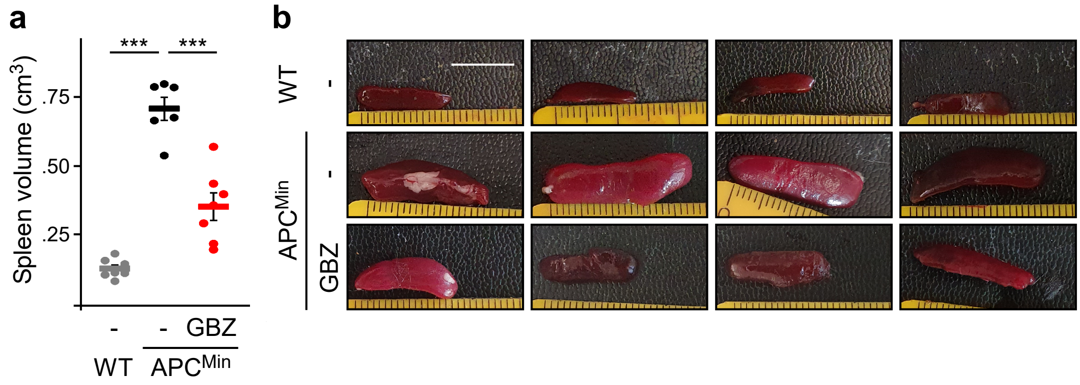

Supplementary Fig. 16 GBZ treatment rescues splenomegaly in APC^{Min} mice. **a** Spleen volumes of APC^{Min} mice without (n=6, black dots) and with (n=7, red dots) GBZ treatment, and of WT littermates, as controls (n=7, grey dots). Results are mean +/- SEM. ***p<0.001 (Two-sided Student's *t*-test). **b** Representative images of spleens from APC^{Min} mice without and with GBZ treatment, and of WT littermates. Scale bar: 1 cm. Source data and exact p-values are provided as source data file.

Supplementary References

1. Soundararajan M*, et al.* Structural diversity in the RGS domain and its interaction with heterotrimeric G protein alpha-subunits. *Proc Natl Acad Sci U S A* **105**, 6457-6462 (2008).

2. Bernkopf DB, Bruckner M, Hadjihannas MV, Behrens J. An aggregon in conductin/axin2 regulates Wnt/beta-catenin signaling and holds potential for cancer therapy. *Nat Commun* **10**, 4251 (2019).

3. Sievers F*, et al.* Fast, scalable generation of high-quality protein multiple sequence alignments using Clustal Omega. *Molecular systems biology* **7**, 539 (2011).

4. Hunt TW, Fields TA, Casey PJ, Peralta EG. RGS10 is a selective activator of G alpha i GTPase activity. *Nature* **383**, 175-177 (1996).

5. Anvarian Z*, et al.* Axin cancer mutants form nanoaggregates to rewire the Wnt signaling network. *Nat Struct Mol Biol*, (2016).

6. Tang Z, Li C, Kang B, Gao G, Li C, Zhang Z. GEPIA: a web server for cancer and normal gene expression profiling and interactive analyses. *Nucleic acids research* **45**, W98-W102 (2017).

7. Cancer Genome Atlas N. Comprehensive molecular characterization of human colon and rectal cancer. *Nature* **487**, 330-337 (2012).

8. Consortium GT. The Genotype-Tissue Expression (GTEx) project. *Nat Genet* **45**, 580-585 (2013).
