# Supplementary material for: Gαi2-induced conductin/axin2 condensates inhibit Wnt/β-catenin signaling and suppress cancer growth
Source: Nat Commun. 2022 Feb 3;13:674. doi: 10.1038/s41467-022-28286-9 (PMC8814139; doi:10.1038/s41467-022-28286-9)
Supplement: Supplementary file 3 — Description of Additional Supplementary Files [file 41467_2022_28286_MOESM3_ESM.pdf]

Description of Additional Supplementary Files

File Name: Supplementary Movie 1
Description: Live Monitoring of GBZ-induced formation of conductin condensates. Time-laps microscopy of GFP fluorescence in U2OS cells stably expressing GFP-tagged conductin for 96 minutes after treatment start with 100 µM GBZ.
