# Supplementary material for: Gαi2-induced conductin/axin2 condensates inhibit Wnt/β-catenin signaling and suppress cancer growth
Source: Nat Commun. 2022 Feb 3;13:674. doi: 10.1038/s41467-022-28286-9 (PMC8814139; doi:10.1038/s41467-022-28286-9)
Supplement: Supplementary file 5 — Reporting Summary [file 41467_2022_28286_MOESM5_ESM.pdf]

nature research

Corresponding author(s): Dominic B. Bernkopf

Last updated by author(s): Dec 20, 2021

Reporting Summary

Nature Research wishes to improve the reproducibility of the work that we publish. This form provides structure for consistency and transparency in reporting. For further information on Nature Research policies, see our Editorial Policies and the Editorial Policy Checklist.

Statistics

For all statistical analyses, confirm that the following items are present in the figure legend, table legend, main text, or Methods section.

n/a	Confirmed	
☐	☒	The exact sample size (*n*) for each experimental group/condition, given as a discrete number and unit of measurement
☐	☒	A statement on whether measurements were taken from distinct samples or whether the same sample was measured repeatedly
☐	☒	The statistical test(s) used AND whether they are one- or two-sided<br>*Only common tests should be described solely by name; describe more complex techniques in the Methods section.*
☒	☐	A description of all covariates tested
☐	☒	A description of any assumptions or corrections, such as tests of normality and adjustment for multiple comparisons
☐	☒	A full description of the statistical parameters including central tendency (e.g. means) or other basic estimates (e.g. regression coefficient) AND variation (e.g. standard deviation) or associated estimates of uncertainty (e.g. confidence intervals)
☐	☒	For null hypothesis testing, the test statistic (e.g. *F*, *t*, *r*) with confidence intervals, effect sizes, degrees of freedom and *P* value noted<br>*Give P values as exact values whenever suitable.*
☒	☐	For Bayesian analysis, information on the choice of priors and Markov chain Monte Carlo settings
☒	☐	For hierarchical and complex designs, identification of the appropriate level for tests and full reporting of outcomes
☐	☒	Estimates of effect sizes (e.g. Cohen's *d*, Pearson's *r*), indicating how they were calculated

Our web collection on statistics for biologists contains articles on many of the points above.

Software and code

Policy information about availability of computer code

Data collection	Bio-Rad CFX Manager 3.0 (qRT-PCR), CellQuest 3.3 (FACS), E.A.S.Y. Win32 4.00.233 (Cell colony images), GeneSys 1.7.0.0 (WB), ImageReader LAS-3000 2.0 (WB), MetaMorph 6.2r4 (IF), Micro-Manager 1.4.22 (Live cell imaging), MikroWin 2000 4.29 (Reporter assay), SoftMax Pro v.5.4.1 (Reporter assay, MTT assay), SWISS-MODEL (RGS structure model), Tecan i-control 2.0 (BODIPY-FL-GTP GAP assay)
Data analysis	Aida Image Analyzer v.3.52 (WB, 2D densitometry), CellQuest 3.3 (FACS), Clustal Omega 1.2.4 (Alignments), Fiji ImageJ 1.53f51 (WB, 2D densitometry), GEPIA (analysis of RNA expression data), MetaMorph 6.2r4 (IF, cell colonies, tumors), Microsoft Excel 14.0.7232.5000, PyMOL 1.8. (Protein structures), Icy 2.2.1.0 (Image-based analysis)

For manuscripts utilizing custom algorithms or software that are central to the research but not yet described in published literature, software must be made available to editors and reviewers. We strongly encourage code deposition in a community repository (e.g. GitHub). See the Nature Research guidelines for submitting code & software for further information.

Data

Policy information about availability of data

All manuscripts must include a data availability statement. This statement should provide the following information, where applicable:

- Accession codes, unique identifiers, or web links for publicly available datasets
- A list of figures that have associated raw data
- A description of any restrictions on data availability

The crystal structure data used in this study are available in the PDB database under accession codes 1DK8 (https://www.rcsb.org/structure/1DK8), 1EMU (https://www.rcsb.org/structure/1EMU), 2GTP (https://www.rcsb.org/structure/2GTP), 2IHB (https://www.rcsb.org/structure/2IHB), 2IK8 (https://www.rcsb.org/structure/2IK8) and 2ODE (https://www.rcsb.org/structure/2ODE). The human cancer data used in this study are available in the The NCI Genomic Data Commons Data Portal database under accession codes COAD (https://portal.gdc.cancer.gov/projects/TCGA-COAD) and READ (https://portal.gdc.cancer.gov/projects/TCGA-

READ). The other mRNA expression data used in this study are available in the GTEx Portal database under accession codes colon-sigmoid (https://www.gtexportal.org/home/eqtls/tissue?tissueName=Colon_Sigmoid) and colon-transverse (https://www.gtexportal.org/home/eqtls/tissue?tissueName=Colon_Transverse).
The source data underlying Figs. 1d-f, 2a-f, h-k, 3d-g, 4b-l, 5d, e, g, h, 6b-j, 7b, d-j, 8a-c, e-g, i, and Supplementary Figs. 2c-e, g, h, j, k, 3b, c, 4a-e, g, 5a, c-e, 6c-e, 7a-l, 8b-k, 9a-e, 11a-g, 12a, b, f, 13a-j, 14a-f and 16a are provided as a Source Data file. All the other data supporting the findings of this study are available within the article and its Supplementary Information.

Field-specific reporting

Please select the one below that is the best fit for your research. If you are not sure, read the appropriate sections before making your selection.

☒ Life sciences ☐ Behavioural & social sciences ☐ Ecological, evolutionary & environmental sciences

For a reference copy of the document with all sections, see nature.com/documents/nr-reporting-summary-flat.pdf

Life sciences study design

All studies must disclose on these points even when the disclosure is negative.

Sample size	For the in vivo experiments, the number of mice was calculated a priori based on expected relevant biological differences using the GPower 3.1 software. These calculations were approved by the governmental authorities who authorized the animal studies (Regierung Unterfranken). For the in vitro experiments, sample sizes between three and six were chosen depending on the differences in exploratory experiments and the variability of the assay. The sample sizes are within the range found in published literature with similar methodologies.
Data exclusions	No data of decent experimental quality was excluded.
Replication	All experimental in vitro findings were reproduced in form of at least three independent biological replicates. The animal studies with APC Min mice and BALB/c-Nude mice were performed once to minimize the number of animals, yet obtaining statistically meaningful results, following the 3Rs rule (replacement, reduction, refinement) for performing animal experiments.
Randomization	APC Min mice were allocated randomly in treatment and control groups. BALB/c-Nude mice were allocated in treatment and control groups according to initial tumor sizes, to guarantee a comparable average tumor burden in both groups at the beginning. For cell culture experiments, samples, i.e. seeded cells, were allocated randomly into experimental groups.
Blinding	For most experiments blinding was unnecessary due to automated objektive readout. The investigator was blinded with respect to the samples for the following experiments: Figs. 2d, e, h, 3d, e, 6b, 8e, f, g, i (measuring tumor sizes of APC Min mice), and Supplementary Figs. 5a, 11e, 12a.

Reporting for specific materials, systems and methods

We require information from authors about some types of materials, experimental systems and methods used in many studies. Here, indicate whether each material, system or method listed is relevant to your study. If you are not sure if a list item applies to your research, read the appropriate section before selecting a response.

Materials & experimental systems

n/a	Involved in the study
☐	☒ Antibodies
☐	☒ Eukaryotic cell lines
☒	☐ Palaeontology and archaeology
☐	☒ Animals and other organisms
☒	☐ Human research participants
☒	☐ Clinical data
☒	☐ Dual use research of concern

Methods

n/a	Involved in the study
☒	☐ ChIP-seq
☐	☒ Flow cytometry
☒	☐ MRI-based neuroimaging

Antibodies

Antibodies used	anti alpha-tubulin (Serotec, MCA77G, YL1/2)<br>anti alpha-tubulin (Sigma-Aldrich, T6199)<br>anti axin2 (CellSignaling, 2151S, 76G6)<br>anti beta-actin (Sigma-Aldrich, A5441, AC-15)<br>anti beta-catenin (Santa Cruz Biotechnologies, sc-7963, E-5)<br>anti Flag (Sigma-Aldrich, F7425)<br>anti G alpha i1 (Santa Cruz Biotechnologies, sc-13533)<br>anti G alpha i2 (Santa Cruz Biotechnologies, sc-13534)<br>anti G alpha i3 (Merck, 371726)<br>anti GFP (Roche, 11814460001, 7.1, 13.1)

anti GFP (GeneTex, GTX113617)
anti GST (CellSignaling, 2624S, 26H1)
anti HA (Sigma-Aldrich, H6908)
anti HA (Roche, 11867423001)
anti RGS-His (Qiagen, 34650)

anti mouse-Cy3 (Jackson ImmunoResearch, 115-165-146)
anti mouse-HRP (Jackson ImmunoResearch, 115-035-146)
anti rabbit-Cy3 (Jackson ImmunoResearch, 111-165-144)
anti rabbit-HRP (Jackson ImmunoResearch, 111-035-144)
anti rat-HRP (Jackson ImmunoResearch, 112-035-143)

Validation

Detection of endogenous proteins:
anti alpha-tubulin (Serotec, MCA77G, YL1/2)
Statement manufacturer's website: "This product has been reported to work in the following applications. This information is derived from testing within our laboratories, peer-reviewed publications or personal communications from the originators." Target species: includes human, verified application: includes Western Blotting.
anti alpha-tubulin (Sigma-Aldrich, T6199)
Statement manufacturer's website: "The antibody is specific for α-tubulin in immunoblotting assays and may be used for localization of α-tubulin in cultured cells or tissue sections."
anti axin2 (CellSignaling, 2151S, 76G6)
The antibody signal was absent after axin2/conductin knockout (Fig. 4l) and reduced by axin2/conductin knockdown (Fig. 4j).
anti beta-actin (Sigma-Aldrich, A5441, AC-15)
Statement manufacturer's website: Product Specification: WB-Cell Line/Tissue Extract: HS-68 Human Fibroblast
anti beta-catenin (Santa Cruz Biotechnologies, sc-7963, E-5)
Statement manufacturer's website: "β-catenin Antibody (E-5) is a high quality monoclonal beta-catenin antibody (also designated beta-Catenin or CTNNB1 antibody) suitable for the detection of the beta-catenin protein of mouse, rat and human origin."
The antibody signal was reduced in SW480 cells by conductin-promoted degradation of beta-catenin in immunofluorescence (Fig. 4a, b) and Western blot based experiments (Fig. 6d, e).
anti G alpha i1 (Santa Cruz Biotechnologies, sc-13533)
anti G alpha i2 (Santa Cruz Biotechnologies, sc-13534)
anti G alpha i3 (Merck, 371726)
The antibody signal for the G alpha i proteins 1, 2 and 3 was abolished by specific shRNA-mediated knockdown of the respective G alpha i protein (Supplementary Fig. 5d).

Detection of protein tags:
anti Flag (Sigma-Aldrich, F7425)
Bands of correct molecular weight were detected (WB) and this specific signal was absent without expression of the tagged proteins (Fig. 4c Flag-Cdt).
anti GFP (Roche, 11814460001, 7.1, 13.1)
Bands of correct molecular weight were detected (WB) and this specific signal was absent without the tagged proteins (Fig. 1f).
anti GFP (GeneTex, GTX113617)
Bands of correct molecular weight were detected (WB) and this specific signal was absent without the tagged proteins (Fig. 2a).
anti GST (CellSignaling, 2624S, 26H1)
Statement manufacturer's website: "Specificity / Sensitivity: GST-Tag (26H1) Mouse mAb detects transfected glutathione S-transferase (GST) fusion proteins." Application: includes Western Blotting.
anti HA (Sigma-Aldrich, H6908)
Bands of correct molecular weight were detected (WB) and this specific signal was absent without the tagged proteins (Fig. 4c HA-Cdt).
anti HA (Roche, 11867423001)
Bands of correct molecular weight were detected (WB) and this specific signal was absent without the tagged proteins (Fig. 2a).
anti RGS-His (Qiagen, 34650)
Bands of correct molecular weight were detected (WB) and this specific signal was absent without the tagged proteins (Supplementary Fig. 4e).

All primary antibodies are commercially available, and valid for the described applications according to statements by the supplier and references at the supplier's homepage.

Eukaryotic cell lines

Policy information about cell lines

Cell line source(s)	Source of all cell lines (DLD1, HEK293, HEK293T, U2OS, SW480): ATCC.
Authentication	Cells were authenticated based on cell morphology and cell size, and indicative features such as e.g. truncated APC in SW480 and DLD1 cells. No further authentication of cell lines was performed.
Mycoplasma contamination	All cell lines were tested negative for mycoplasma contamination.
Commonly misidentified lines (See ICLAC register)	None of the used cell lines is commonly misidentified according to the ICLAC register.

Animals and other organisms

Policy information about studies involving animals; ARRIVE guidelines recommended for reporting animal research

Laboratory animals	In this study female CAnN.Cg-Foxn1nu/Crl and C57BL/6J-ApcMin/J mice were used. The CAnN.Cg-Foxn1nu/Crl mice were seven weeks old when we started the experiment by injecting the tumor cells, and about 12 weeks old when the experiment was terminated.<br>The C57BL/6J-ApcMin/J mice were about 6 weeks old when we started the GBZ treatment, and about 15 weeks when the experiment was terminated.
Wild animals	No wild animals were used in the study.
Field-collected samples	No field-collected samples were used in the study.
Ethics oversight	An application for the animal study was approved by governmental authorities (Regierung Unterfranken; 55.2.2-2532.2-923) according to the German Tierschutzgesetz. The ethical reasonableness of the study was critically assessed by the authorities and/or their expert advisors before approval.

Note that full information on the approval of the study protocol must also be provided in the manuscript.

Flow Cytometry

Plots

Confirm that:

☒ The axis labels state the marker and fluorochrome used (e.g. CD4-FITC).

☒ The axis scales are clearly visible. Include numbers along axes only for bottom left plot of group (a 'group' is an analysis of identical markers).

☒ All plots are contour plots with outliers or pseudocolor plots.

☒ A numerical value for number of cells or percentage (with statistics) is provided.

Methodology

Sample preparation	Cells from cell culture were trypsinized and colltected in FACS buffer.
Instrument	FACSCalibur, BD Bioscience
Software	CellQuest 3.3, BD Bioscience
Cell population abundance	The abundance of the R-FlincA positive population was about 10% of the cells. In case of untransfected control cells, only 10 cells appeared in R2 in contrast to 4202 cells for cells transiently expressing R-FlincA, suggesting that our gating for R-FlincA positive cells is stringent and the population is very pure. However, purity of the fraction was not formally quantified, e.g. by re-sorting.
Gating strategy	Gating R1: Gating based on forward scatter (FSC) and side scatter (SSC) was performed to exclude cell debris. The boundary between cell debris (low FSC and SSC height) and cells to include in the analysis (increased FSC and SSC height) was indicated by an area with low signal intensity between both populations. A figure exemplifying the gating is provided (Supplementary Fig. 7n).<br>Gating R2: Gating for R-FlincA positive cells (Supplementary Fig. 7m). Parallel FACS analysis of untransfected control cells was used to identify the population of R-FlincA negative cells. The gate R2 for R-FlincA positive cells was positioned just adjacent to this negative population towards the right side to include all cells with a higher FL3 fluorescence.

☒ Tick this box to confirm that a figure exemplifying the gating strategy is provided in the Supplementary Information.
